# Supplementary figures and images for: Effect of irrigation with treated wastewater on bermudagrass (Cynodon dactylon (L.) Pers.) production and soil characteristics and estimation of plant nutritional input
Source: PLoS One. 2022 Jul 15;17(7):e0271481. doi: 10.1371/journal.pone.0271481 (PMC9286233; doi:10.1371/journal.pone.0271481)

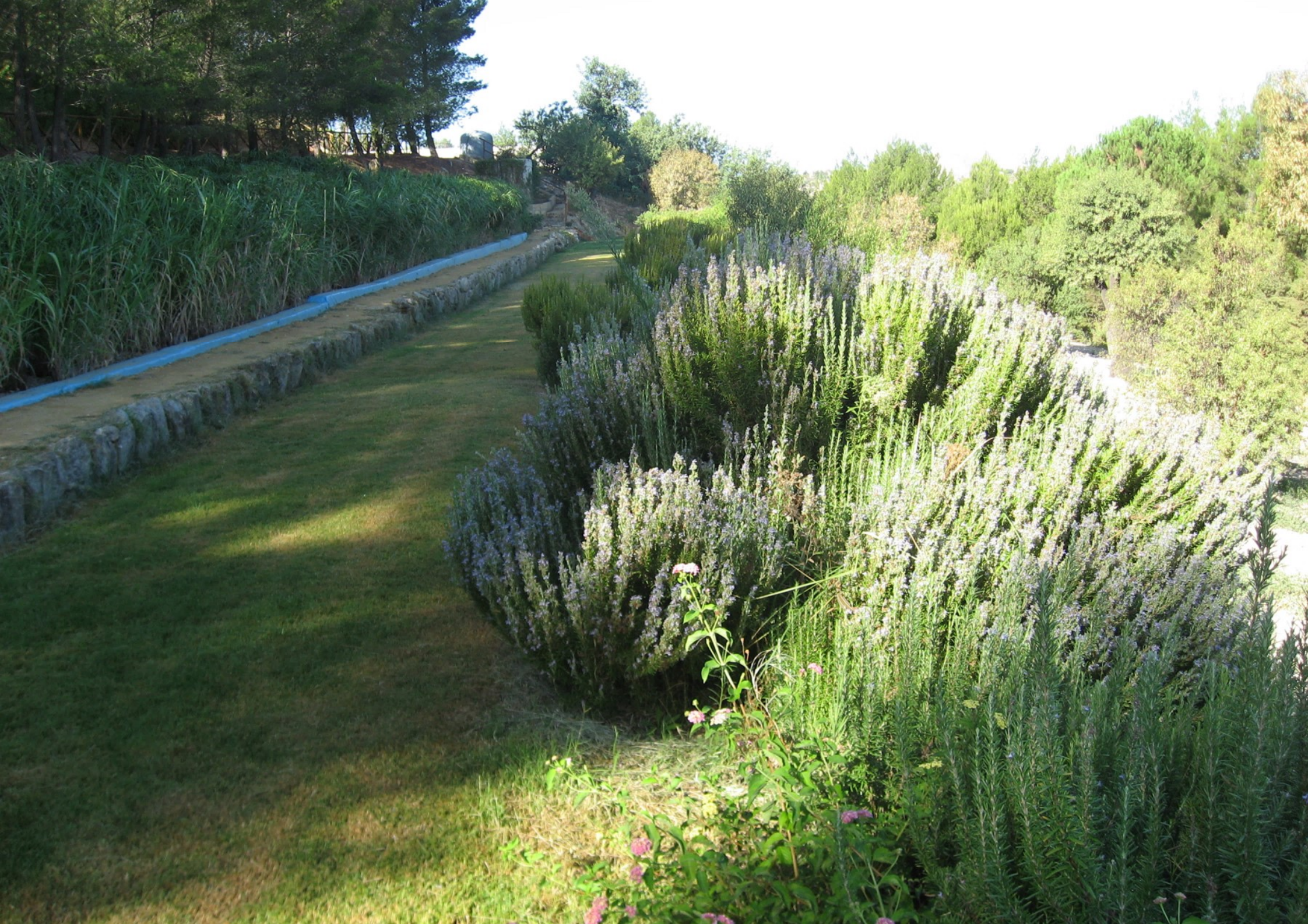

Supplement: S1 Fig — (PDF) [file pone.0271481.s001.pdf]

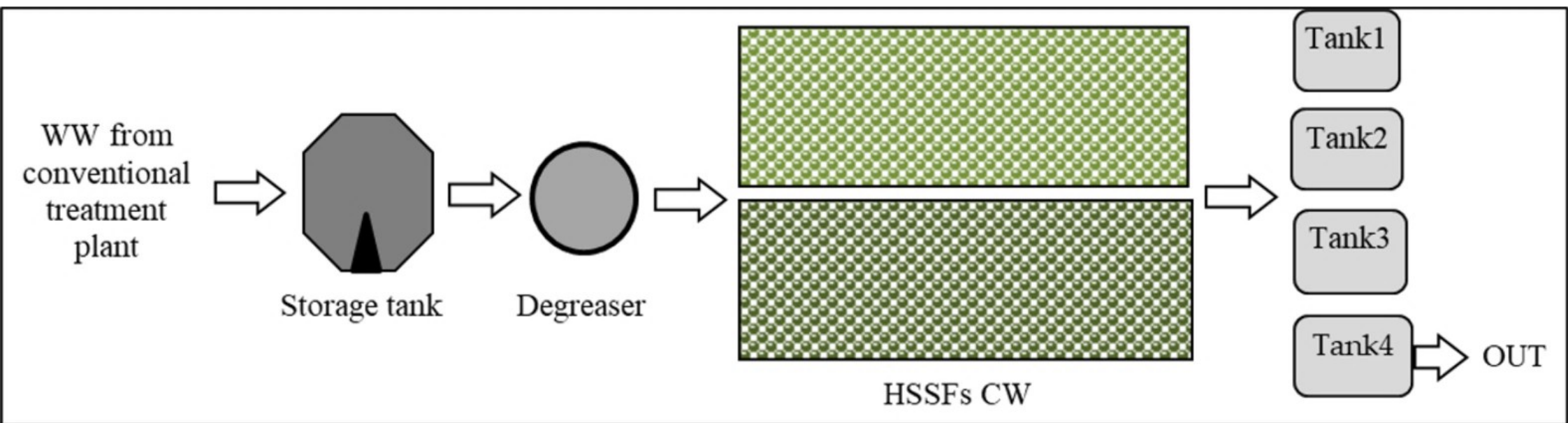

Supplement: S2 Fig — (PDF) [file pone.0271481.s002.pdf]
