# Supplementary material for: Effect of irrigation with treated wastewater on bermudagrass (Cynodon dactylon (L.) Pers.) production and soil characteristics and estimation of plant nutritional input
Source: PLoS One. 2022 Jul 15;17(7):e0271481. doi: 10.1371/journal.pone.0271481 (PMC9286233; doi:10.1371/journal.pone.0271481)
Supplement: Attachment — Submitted filename: Reviewer comments-Plos One.docx [file pone.0271481.s009.docx]

**Reviewer comments**

The manuscript entitled ‘Irrigation with treated wastewater affects biomass production of bermudagrass and reduces the need for plant nutrients’ is an interesting research work. The article is not well organized with robust bibliography which consists of enough data and enough scientific fundamental conceptualization. The article is not suitable for publication in PLOSONE.

- Title of the manuscript need to be improved. In the present form it appears as the statement. Authors may consider inclusion of scientific name of bermudagrass in brackets ().
- Authors are advised to reduce the data values (large values) to single decimal place throughout the manuscript. In abstract, please check, …….29.00 kg P ha^-1^ year^-1^, I think should be K rather than P????
- Authors have written several abbreviations e.g. BOD, COD, TN, TP etc, which needs to written in full at their first appearance in the text.
- In abstract authors have written that soil salinity increased significantly…., they need to highlight the statistical significance e.g. *p<*0.05 or *p*<0.01.
- The introduction has citation of 32 references, of which only 5-6 references are latest one (work published after 2019). Authors are advised to cite recent references.
- The major drawback of the introduction section is the lack of state-of the art of the research works and its connection with the problem formulation, research objectives and the hypothesis. Authors should critically work on that.
- Authors need to specify the amount of wastewater for irrigation per hectare area or number of irrigations to be given with wastewater per annum. It is written in conclusion that application of TWW irrigation in medium-term leads to increase in bermudagrass turf biomass yields. Clarify in conclusion what does medium term means. Conclusion must be clear.
- Figure 1 and 2 are needless.
- The quality of figures 4 and 5 is very bad. It is difficult to read the legends.
- Figure 6 lacks statistical analysis
